# Oleuropein Aglycone Modulates Oxidative Stress and Autophagy‐Related Pathways in Human Skeletal Muscle Cells

**DOI:** 10.1002/biof.70058

**Published:** 2025-11-19

**Authors:** Giulia Polacchini, Andrea Venerando, Anne Bigot, Monica Colitti

**Affiliations:** ^1^ Department of Agricultural, Food, Environmental and Animal Sciences University of Udine Udine Italy; ^2^ Sorbonne Université, Inserm, Institut de Myologie, Centre de Recherche en Myologie Paris France

**Keywords:** aging, human skeletal muscle cells, oleuropein aglycone, ROS

## Abstract

A gradual loss of muscle mass and strength increases the risk of falls, frailty and mortality. It is the result of a combination of intrinsic factors such as oxidative stress, mitochondrial dysfunction and extrinsic factors such as poor nutrition and inactivity. Oleuropein aglycone (OLE), a compound extracted from olive leaves, combats oxidative damage through its antioxidant and autophagy‐inducing properties. It activates AMPK and FOXO3a signaling pathways, autophagy, mitochondrial function and muscle health. OLE is investigated in the human immortalized myoblast cell line AB1079 for its protective effect against muscle oxidative stress. Oxidative stress was induced in the AB1079 cell line after 7 days of differentiation by hydrogen peroxide (H_2_O_2_), leading to a significant increase in reactive oxygen species formation, which was reduced by approximately 43% by pretreatment with OLE. Cells treated with H_2_O_2_ showed a 33% increase in stress‐induced senescent cells, while pretreatment with OLE significantly reduced the stained area of the X‐gal reaction by 12% compared to H_2_O_2_. OLE increased the expression of genes involved in antioxidant defense and influencing the autophagic process by inducing an oscillator AMPK phosphorylation, as well as the expression of the stress‐induced metabolic regulators SESN2 and SESN3. OLE has been shown to counteract the oxidative environment and promote autophagy‐related signaling in vitro, suggesting a potential role in preventing cellular mechanisms associated with muscle aging. Further in vivo studies are required to confirm functional anti‐aging effects.

## Graphical abstract

Oleuropein aglycone activates AMPK/FOXO signaling in skeletal muscle cells, enhancing antioxidant defenses and autophagy. In summary, it improves cellular redox balance and protects muscle cells from stress‐induced early senescence.

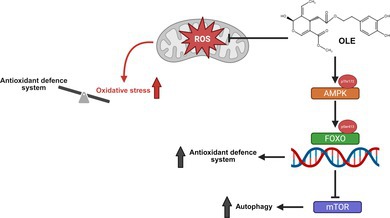

**Abbreviations**
AKT: protein kinase B
AMPK: AMP‐activated protein kinase
DCFH‐DA: 2',7'‐dichlorofluorescein diacetate
DMSO: dimethyl sulphoxide
FOXO3a: Forkhead box O3A
H_2_O_2_
: hydrogen peroxide
LC3B: microtubule associated protein 1 light chain 3 beta
mTOR: mechanistic target of rapamycin
NRF2: nuclear factor erythroid 2‐related factor 2
OLE: oleuropein aglycone
PPARGC1A: peroxisome proliferator‐activated receptor gamma coactivator 1‐alpha
ROS: reactive oxygen species
SA‐β‐gal: SA‐β‐galactosidase
SESN1: sestrin 1
SESN2: sestrin 2
SESN3: sestrin 3
SOD2: superoxide dismutase 2
Ulk1: Unc‐51 like autophagy activating kinase 1

## 1 Introduction

In the elderly, the gradual decline in muscle mass and strength generally emphasizes a slow and progressive skeletal muscle disease in which the loss of muscle mass and strength is accompanied by functional decline of the musculoskeletal system that in turn exposes the geriatric population to an increased risk of falls with fractures, obesity, frailty, hospitalization and mortality. Defining muscle weakness or sarcopenia is challenging due to its multifactorial nature. In fact, both intrinsic factors such as the release of proinflammatory cytokines, oxidative stress and reduced mitochondrial function [1] and extrinsic factors such as nutritional status, drug use and lack of physical activity [2] contribute to the development and progression of sarcopenia in a complex and dynamic relationship. For this reason, it is critical to understanding cellular pathways that may contribute to aging and related pathologies.

During muscle contraction large amounts of reactive oxygen species (ROS) are produced, which make skeletal muscles more susceptible to oxidative stress and cellular senescence [3]. In older people, it is hypothesized that muscle aging or disease is actually associated with high levels of ROS and contractile dysfunction possibly due to chronic muscle inactivity [4]. In fact, increased production of endogenous oxidants was observed in isolated skeletal muscle fibers from older mice compared to younger mice at rest [5]. There is ample evidence that oxidative damage contributes to the aging process and deterioration of muscle performance. This has led to extensive research into the efficacy of antioxidants as a potential therapeutic approach to mitigate the damaging effects of free radicals associated with muscle aging [6]. Oleuropein is a bioactive phenolic compound extracted from olive leaves that is well known for its antioxidant and anti‐inflammatory potential [7, 8]. The pharmacological properties of this natural compound are largely attributed to its strong antioxidant activity [9], which is due to its ability to quench the reactivity of free radicals by forming hydrogen bonds between the hydroxyl groups within the molecule and the phenoxy radicals [10]. Interestingly, oleuropein can act as an antioxidant on both a preventive and interventional level. Studies in animals and cell cultures have shown that oleuropein aglycone increases the autophagy response through the activation of 5′‐AMP‐activated protein kinase (AMPK) and the expression of markers associated with the autophagy‐regulating mechanistic target of rapamycin (mTOR) protein and the transcription factor Forkhead box O3A (FOXO3a) [11, 12]. Under stress conditions, AMPK activates autophagy by either inhibiting mTORC1 [13] or activating Ulk1 for phosphorylation [14]. Indeed, high mTORC1 activity has been shown to cause damage to skeletal muscle fibers in older mice and humans [15]. In addition, AMPK can directly phosphorylate the transcription factor FOXO3a (at Ser413), which increases its activity and promotes its nuclear translocation [16]. At the nuclear level, FOXO3a switches on target genes that support cellular antioxidant defense strategies by preventing the formation of ROS, scavenging already formed ROS (e.g., superoxide dismutase and catalase), and promoting adaptation to the oxidative environment by stimulating signaling pathways that activate the protective response. In addition, FOXO3a activates genes, such as peroxisome proliferator‐activated receptor gamma coactivator 1‐alpha (PPARGC1A), creating a feedback loop that improves mitochondrial function and antioxidant capacity, as well as genes encoding proteins such as sestrins, which are involved in the antioxidant response, autophagy and age‐related diseases [17, 18]. Interestingly, FOXO‐dependent production of sestrin 3 has been shown to inhibit mTOR complex 1 [19].

Conversely, antagonistic regulation of FOXO3a by protein kinase B (Akt) in response to stimulation by insulin/growth factors leads to direct phosphorylation of FOXOs, which are translocated to the cytoplasm and inactivated [20].

In this study, the effects of oleuropein aglycone (OLE) on oxidative stress responses and autophagy were investigated in a human myoblast model. These findings are limited to an in vitro system, and further in vivo studies are required to determine whether these mechanisms translate into functional anti‐aging effects at the organismal level.

## 2 Material and Methods

### 2.1 Chemicals and Reagents

Oleuropein aglycone (OLE) was purchased from CliniSciences Group (Nanterre, France) and stored in DMSO (EuroClone S.p.A., Milan, Italy). Rapamycin and fluorescent probe 2′,7′‐dichlorofluorescein diacetate (DCFH‐DA) were acquired from Sigma‐Aldrich (Merck KGaA, Darmstadt, Germany). Geltrex solution was purchased from Life Technologies (Thermo Fisher Scientific Inc., MA, USA).

### 2.2 Cell Culture and Treatments

Immortalized myogenic cell lines are an important tool for skeletal muscle research and are used as therapeutically relevant models in various basic studies on muscle cell function. The human immortalized myoblast cell line AB1079 was obtained from the MyoLine immortalisation platform of the Institut de Myologie (Paris, France). Cells were harvested from a muscle biopsy of the quadriceps of a healthy male subject aged 38 years, obtained by the EuroBioBank Myobank‐AFM, with the subject's agreement based on an informed consent form and anonymisation prior to immortalisation, in accordance with the EU GDPR regulation. The cells were immortalized using hTERT/cdk4 as described in Mamchaoui et al. [21].

Immortalized myoblasts were cultivated in a growth medium composed of 199 medium (Invitrogen, Waltham, MA, USA) and DMEM high‐glucose (Invitrogen, Waltham, MA, USA) at a 1:4 ratio, supplemented with 20% FBS (Sigma, Saint Louis, MO, USA), 50 μg/mL gentamicin, 25 μg/mL bovine fetuin, 5 ng/mL hEGF, 0.5 ng/mL bFGF (Life Technologies, Carlsbad, CA, USA), 0.2 μg/mL dexamethasone and 5 μg/mL human insulin (Merck KGaA, Darmstadt, Germany). Cells were seeded on Geltrex‐coated supports and maintained in a humidified atmosphere of 5% CO_2_ at 37°C. Differentiation was induced at 80% cell confluence by replacing the growth medium with DMEM high‐glucose supplemented with 50 μg/mL gentamicin and 10 μg/mL insulin (Merck KGaA, Darmstadt, Germany) for 7 days.

After 7 days of differentiation, cells were treated with different concentrations of OLE (10, 25 or 50 μM) and H_2_O_2_ (50, 100, 200 or 300 μM) in 0.05% DMSO to determine the best concentration to use in the experimental setup without affecting cell viability. Vehicle alone (0.05% DMSO) was used as a control. In the experimental design, AB1079 cells were pretreated with OLE for 24 h and exposed to H_2_O_2_ for the last 2 h to promote a stress‐induced early senescence and to observe the preventive role of OLE (OLE + H_2_O_2_) [22].

To detect autophagy cells were incubated for 24 h with 500 nM rapamycin, which was used as a positive control.

### 2.3 Cell Viability Assay

Cell viability was determined using the 3‐(4,5‐dimethylthiazol‐2‐yl)‐2,5‐diphenyltetrazolium bromide (MTT) assay. AB1079 cells were seeded in Geltrex‐coated 96‐well plate (2 × 10^4^ cells/well) differentiated for 7 days and treated as indicated. After treatment, cells were rinsed with phosphate buffered saline (PBS) and incubated with 5 mg/mL MTT in PBS for 4 h at 37°C. The produced formazan was dissolved in DMSO and incubated overnight (O/N) at 37°C. Absorbance was measured at *λ* = 570 nm by Spark microplate reader (Tecan Group Ltd., Switzerland).

### 2.4 Measurement of Total Intracellular ROS Levels

ROS were estimated using the fluorescent probe DCFH‐DA that crosses cell membranes, and it is hydrolyzed to nonfluorescent DCFH by intracellular esterase. In the presence of ROS, DCFH is rapidly oxidized to fluorescent dichlorofluorescin (DCF) that reflects the amount of total intracellular ROS formed.

AB1079 cells were seeded in a Geltrex‐coated 96‐well plate (2 × 10^4^ cells/well), differentiated for 7 days and treated according to the experimental protocol. Prior to H_2_O_2_ treatment, the plates were incubated in the dark with 30 μM DCFH‐DA for 30 min at 37°C. After washing with PBS, the fluorescence intensity was measured at 37°C with an excitation wavelength of 485 nm and an emission wavelength of 530 nm using the Spark microplate reader. Total ROS production was calculated as an increase in fluorescent signal in comparison to the vehicle, while the antioxidant activity was calculated as a reduction in fluorescent signal in comparison to the H_2_O_2_‐treated sample.

### 2.5 SA‐β‐Galactosidase (SA‐β‐Gal) Senescence Assay

Senescence induction and anti‐aging effect were assessed using the SA‐β‐gal staining test, in which the presence of blue colored cells indicates senescent cells. AB1079 cells were seeded in Geltrex‐coated μ‐slide eight‐well ibiTreat chamber at a density of 6 × 10^4^ cells/well, differentiated for 7 days, and finally treated as indicated. After treatment, cells were incubated with 4% paraformaldehyde (PFA) solution for 15 min at room temperature (RT). After two washes with PBS, cells were incubated for 24 h at 37°C without CO_2_ with a freshly prepared SA‐β‐gal staining solution 40 mM (pH 6.0) consisting of 1 μg/mL X‐gal dissolved in 0.1 M citric acid and 0.2 M sodium phosphate, 5 mM potassium ferricyanide, 5 mM potassium ferrocyanide, 150 mM NaCl, and 2 mM MgCl_2_.

The images of the stained cells were acquired with Axio Observer Z1 microscope equipped with the EC‐PLAN Neofluar 20×/0.50 M27 objective and the Infinity Color Corrected System, as well as with the AxioCam 506 camera and Zen blue software (Carl Zeiss, Jena, Germany). The percentage of the blue‐colored area was calculated using ImageJ 1.53r software.

### 2.6 RNA Extraction and Real Time‐PCR

To extract RNA from the AB1079 cell cultures, 0.1 mL cm^−2^ of TRIzol reagent was added in the Petri dishes after culture medium removal. Samples were collected and RNA was extracted using the PureLink RNA Mini Kit (Thermo Fisher Scientific Inc., MA, USA) following the manufacturer's instructions. The concentration of the total RNA extracted was quantified using the acid nucleic quantification tool of the Spark microplate reader. Purity of RNA samples ranged between 1.8 and 1.9.

Primer3 Input software was used to design primers [23] for genes related to antioxidant defense: sestrin 1 (SESN1), sestrin 2 (SESN2), sestrin 3 (SESN3), nuclear factor erythroid 2–related factor 2 (NRF2), peroxisome proliferator‐activated receptor γ coactivator1α (PPARGC1A), and superoxide dismutase 2 (SOD2).

GeneBank accession, primers, product lengths and relative annealing temperatures for each gene are listed in Table 1, according to the HUGO Gene Nomenclature Committee.

**TABLE 1:** Oligonucleotide primer sequences for real time‐PCR analysis.

Gene	GeneBank accession	Primer sets	Product length (bp)	*T*_m_ (°C)
ACTB	NM_001101	Forward: 5′‐CTCTTCCAGCCTTCCTTCCT‐3′	116	59.4
		Reverse: 5′‐AGCACTGTGTTGGCGTACAG‐3′		
B2M	NM_004048	Forward: 5′‐AAGATGAGTATGCCTGCCGT‐3′	97	57.3
		Reverse: 5′‐TCAAACCTCCATGATGCTGC‐3′		
NRF2	NM_006164	Forward: 5′‐GGTTGCCCACATTCCCAAATC‐3′	106	58.0
		Reverse: 5′‐CGTAGCCGAAGAAACCTCA‐3′		
PPARGC1A	NR_148985.2	Forward: 5′‐GCCCAGGTACAGTGAGTCTT‐3′	105	59.4
		Reverse: 5′‐GTGAGGACTGAGGACTTGCT‐3′		
SOD2	DQ003134.1	Forward: 5′‐CTGGAACCTCACATCAACGC‐3′	100	60.0
		Reverse: 5′‐CCTGGTACTTCTCCTCGGTG‐3′		
SESN1	NM_014454.3	Forward: 5′‐CACACATTCAGACCTCCTTC‐3′	161	56.6
		Reverse: 5′‐TGTAACTGCCTCATCTTTTCC‐3′		
SESN2	NM_031459	Forward: 5′‐AGCCTCACCTACAATACCATC‐3′	127	58.0
		Reverse: 5′‐TCACCTCCCCATAATCATAGTC‐3′		
SESN3	NM_144665	Forward: 5′‐AGAAAGCAAACCACAGCCAG‐3′	111	58.0
		Reverse: 5′‐GTTGCAAGTGGACCTGACAG‐3′		

Abbreviations: ACTB, β‐actin; B2M, β‐2‐microglobulin; NRF2, nuclear factor erythroid 2‐related factor 2; PPARGC1A, peroxisome proliferator‐activated receptor γ coactivator1α; SESN1, sestrin1; SESN2, sestrin2; SESN3, sestrin3; SOD2, superoxide dismutase 2; Tm, annealing temperature.

Total RNA was reverse‐transcribed with the ImProm‐II Reverse Transcription System (Promega, Madison, WI) in a MJ thermal cycler model PT‐100 (MJ Research Inc., USA) following the manufacturer's instructions. Real‐time PCR analyses were performed in triplicate for each treatment using Platinum SYBR Green qPCR SuperMix‐UDG reaction kit (Thermo Fisher Scientific Inc., MA, USA).

For each gene, an aliquot of cDNA samples was pooled and standard curves with serial dilution of the pool were used to optimize Real Time‐PCR conditions in terms of cDNA and primer concentrations and to calculate efficiency, fluorescence baseline, and threshold. PCR amplification was achieved by applying 40 cycles (10s at 95°C, 30s at the specific annealing temperature, 30s at 72°C) in a 96‐well spectrofluorometric thermal cycler CFX (Bio‐Rad, Milan, Italy). The analysis of melting curves of the amplification products was performed at the end of each PCR reaction to confirm that a single PCR product was detected.

Target gene expression was normalized using geometric mean values between β‐actin (ACTB) and β‐2‐microglobulin (B2M) mRNAs and analyzed on 7d cells as relative fold change between treatments (H_2_O_2_, OLE and OLE + H_2_O_2_) compared to vehicle [24].

### 2.7 Cyto‐ID Fluorescence Microscopy Autophagy Detection Assay

The induction of autophagy was evaluated by fluorescence microscopy assay using the Cyto‐ID Autophagy Detection Kit 2.0 (Enzo Life Science, USA) containing a fluorescent probe that marks vesicles produced during the autophagic process [25].

Briefly, AB1079 cells were seeded in a μ‐slide 8 well IbiTreat chamber and differentiated for 7 days before being treated as indicated. Rapamycin treatment was used as a positive control of autophagy induction. After treatment, cells were washed with PBS and stained following the manufacturer's protocol. Afterwards, stained cells were washed with PBS and fixed with a 4% PFA solution for 15 min at RT. Images were acquired with the fluorescence microscope Axio Observer Z1 equipped with the EC‐PLAN Neofluar 40×/0.75 M27 objective and Infinity Color Corrected System (ICS), AxioCam and Zen blue software (Carl Zeiss, Jena, Germany). The filters were set to 470/525 nm for the fluorescence probe to visualize the autophagic vesicles and 390/460 nm for the Hoechst 33258 dye to visualize the cell nuclei. The autophagy vesicles were counted using the ImageJ 1.53r software. Autophagy induction is indicated as an increase in the number of vesicles in comparison to the vehicle.

### 2.8 Total Protein Extraction and Western Blot Analysis

After 7 days of differentiation, AB1079 cells were detached from the Petri dishes (Ø 10 cm) with a scraper and collected in PBS. Cells were lysed in ice‐cold RIPA buffer containing protease inhibitor cocktail (Roche, Switzerland), and 1 mM sodium orthovanadate and 1 mM sodium fluoride to inhibit protein phosphatases. Total protein concentration of the samples was determined using the Bradford colorimetric method measuring the absorbance at 595 nm with the Spark microplate reader (Tecan Group Ltd., Switzerland).

Equal amounts of proteins from each sample (40 μg) were separated by Tris‐glycine‐SDS‐PAGE. Then, proteins were transferred to PVDF membranes (Immobilon‐P; Millipore, Darmstadt, Germany) using the semi‐dry Biometra Fastblot apparatus (Analytik Jena GmbH+Co, Jena, Germany). The membranes were incubated overnight at 4°C with primary antibodies diluted in 1% (w/v) bovine serum albumin in Tris‐buffered saline (TBS) with 0.1% (w/v) Tween 20. The primary antibodies were: rabbit anti‐AMPKα (23A3, Cell Signaling), rabbit anti‐p‐AMPKα (Thr172) (40H9, Cell Signaling), mouse anti‐LC3B (SC‐376404, Santa Cruz), rabbit anti‐p16 (10883‐1‐AP, Proteintech), rabbit anti‐p62/SQSTM1 (P0067, Sigma Merck), rabbit anti‐p‐p62/SQSTM1 (Ser403) (14,354, Cell Signaling), mouse anti‐FOXO3a (D7D3Y, Cell Signaling), anti‐p‐FOXO3a (Ser413) (PA5‐104594, Thermo Fisher Scientific), rabbit anti‐p‐FOXO3a (Ser253) (9466, Cell Signaling), rabbit anti‐SESN1 (GTX637400, GeneTex), rabbit anti‐SESN2 (GTX116925, GeneTex) and rabbit anti‐SESN3 (GTX112277, GeneTex). Mouse β‐actin antibody (A2228, Sigma Merck, Darmstadt, Germany) was used as a loading control. After overnight incubation, the membranes were rinsed in 0.1% (w/v) Tween 20 TBS and incubated for 1 h at room temperature with horseradish peroxidase (HRP)‐conjugated secondary antibodies. The HRP‐conjugated secondary antibodies were IgG goat anti‐rabbit IgG (H + L) (170‐6515, BioRad) and IgG goat anti‐mouse IgG (H + L) (170‐6516, BioRad). Chemiluminescence detection of HRP‐conjugated secondary antibodies was performed using G:box chemi XX6/XX9 (Syngene, Cambridge, UK) and quantified using ImageJ 1.53r software.

### 2.9 Statistical Analysis

All results are presented as means ± SD and were analyzed by XLSTAT statistical software [26].

Cell viability and percentage ROS content were obtained from at least eight biological replicates and the ratio of protein expression was determined from five technical replicates for each treatment. Data were compared using one‐way ANOVA with Dunnett's post hoc statistical test in comparison to vehicle or H_2_O_2_‐treated cells. Data from blue X‐gal area and the number of autophagy vesicles were compared using the two‐tailed comparisons Mann–Whitney statistical test.

Gene expression data are presented as relative fold‐change values compared to vehicle and were analyzed using a one‐way ANOVA with Dunnett's statistical test.

## 3 Results

### 3.1 Experimental Setup

**FIGURE 1:**
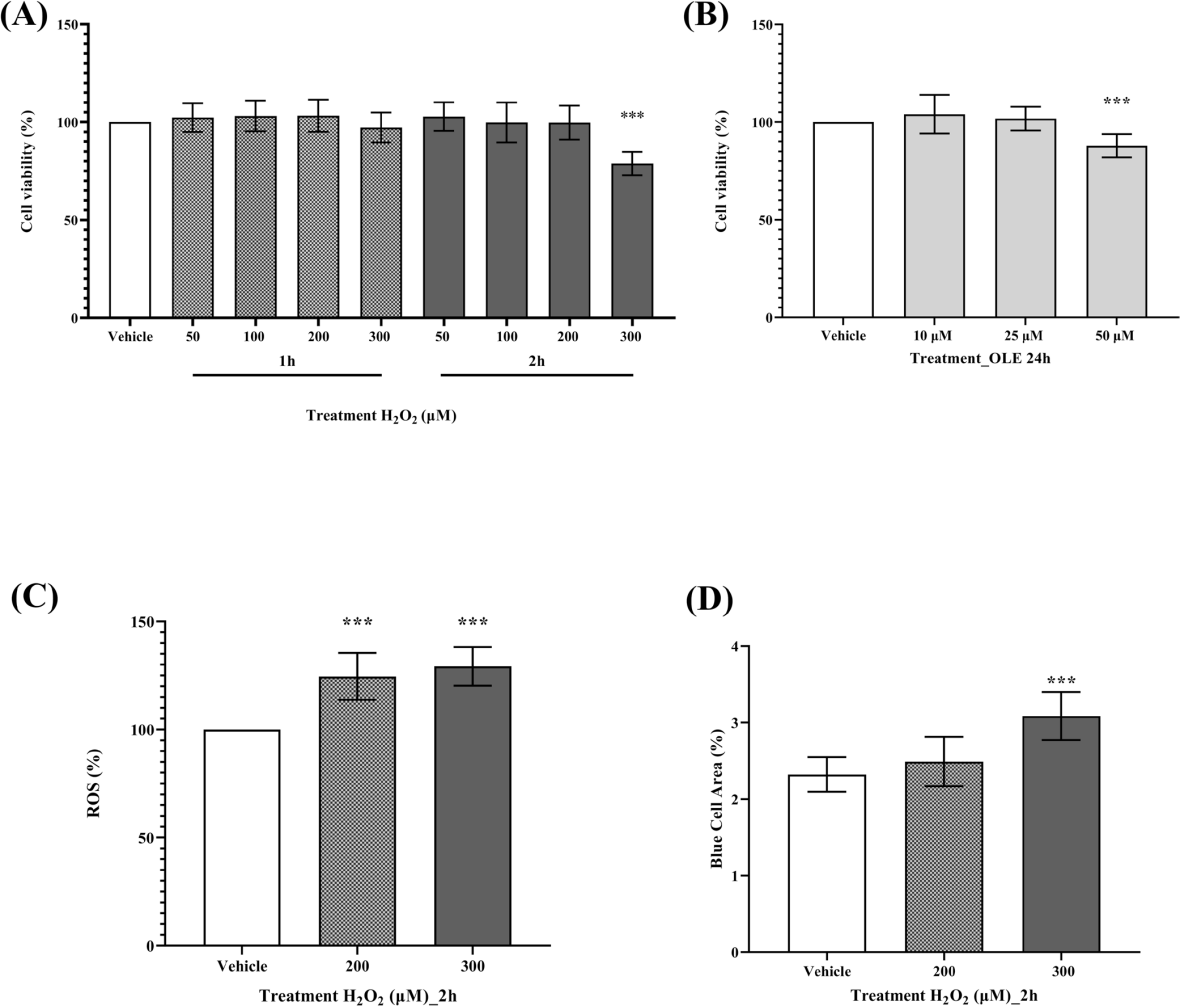
Treatment setup of H_2_O_2_ and OLE on AB1079 cells. (A) Viability of cells treated with vehicle (DMSO 0.05%) or H_2_O_2_ 50, 100, 200 and 300 μM for 1 h or 2 h. (B) Viability of cells treated with OLE 10, 25 and 50 μM for 24 h. (C) The percentage of total ROS and (D) the effect of H_2_O_2_ treatment on stress‐induced premature senescence in cells treated with the vehicle DMSO 0.05%, with H_2_O_2_ 200 μM and 300 μM for 2 h. H_2_O_2_ treatment with 300 μM for 2 h resulted in a significant increase in ROS formation and blue senescent cell area compared to vehicle cells. ***p < 0.0001 compared to vehicle.

Hydrogen peroxide (H_2_O_2_) is a pro‐oxidant model frequently used in in vitro studies. H_2_O_2_ has been shown to induce senescence at high concentrations in the range of 50–300 μM [27, 28]. Studies in human fibroblasts indicate that H_2_O_2_ promotes the accumulation of molecular hallmarks associated with stress‐induced senescence [29, 30, 31]. To setup the dose‐ and time‐dependent pro‐oxidative effect of H_2_O_2_ exposure in differentiated AB1079 cells without major effects on vitality, MTT cell viability assays were performed. Cells were treated with different concentrations of H_2_O_2_ for 1 and 2 h. It should be noted that a significant decline (*p* < 0.0001) in cell viability was observed (% vs. vehicle) only in the case of a 2‐h treatment period with 300 μM H_2_O_2_, with approximately 79% being preserved (Figure 1A). In contrast, lower concentrations of H_2_O_2_ (50–200 μM) or shorter incubation times did not alter cell viability. The protocol involving a 2‐h H_2_O_2_ exposure was designed to evaluate early senescence‐associated responses under acute oxidative stress, whereas chronic senescence induction would require longer post‐treatment culture periods [31]. Moreover, to assess the preventive effect of OLE, cells were collected shortly after H_2_O_2_‐induced oxidative stress. Differentiated AB1079 cells were further tested with OLE alone (10–50 μM) to select the safer concentration to be used in the experimental setup (Figure 1B). Notably, treatment with 50 μM OLE resulted in a significant reduction in the percentage of viable cells in comparison to the untreated control (87.9% ± 6.0%, *p* < 0.0001). Conversely, OLE at a concentration of 10 μM or 25 μM did not alter cell viability (Figure 1B).

To confirm the increase in ROS production after H_2_O_2_ challenge, the percentage of ROS in differentiated AB1079 cells was quantified using the fluorescent probe DCFH‐DA. As expected, ROS production was increased by 25% and 29% after 2 h exposure to 200 and 300 μM H_2_O_2_, respectively (*p* < 0.0001) (Figure 1C).

H_2_O_2_ is the most commonly used trigger for stress‐induced premature senescence in cell models [32]. To determine the most effective concentration of H_2_O_2_ for generating stress‐induced senescence in the AB1079 cell model over a 2‐h period, the SA‐β‐gal enzyme assay was used. It was found that treatment with 300 μM H_2_O_2_ resulted in a significant increase in blue cell area, confirming the senescence induction and thus underpinning the selection of this dose as the optimal dose for subsequent experiments, a decision that was consistent with the results of the viability assays previously performed (Figure 1D).

In view of these results, a 2‐h exposure to 300 μM H_2_O_2_ was chosen in the following experimental setup as an effective challenge to induce oxidative stress in AB1079 without dramatically affecting cell viability. Treatment with 25 μM OLE was selected for the following experiments.

### 3.2 Experimental Design

**FIGURE 2:**
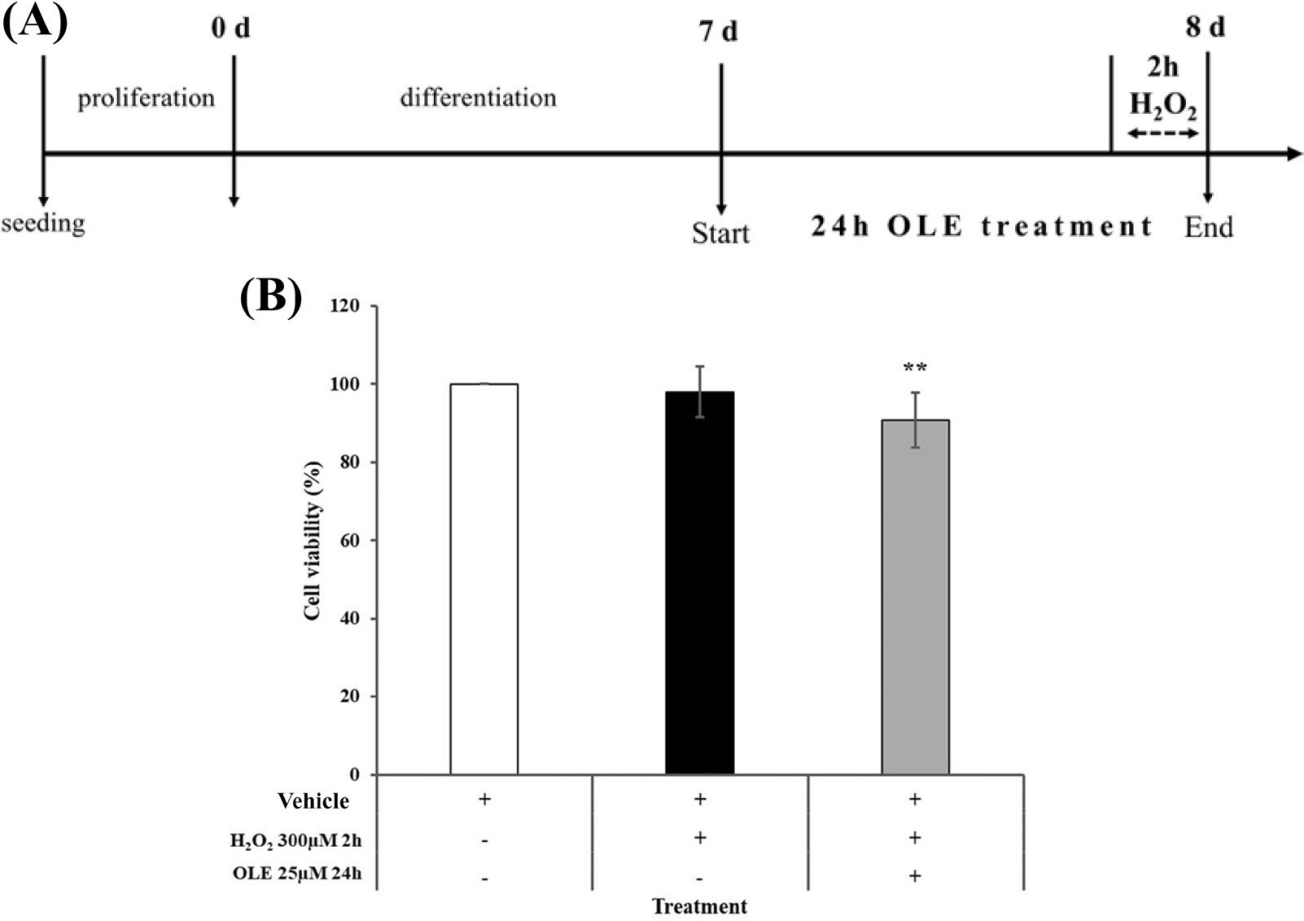
Viability test according to the experimental design in AB1079 cells. (A) The experimental design included cells that were 80% confluent and were differentiated for 7 days. From day 7, OLE treatment started for 24 h and, after 22 h, cells were treated with 300 μM H_2_O_2_ for 2 h to induce oxidative stress and senescence. (B) Cell viability measurements were performed on cells treated according to the experimental setup. Cells were treated with the vehicle (DMSO 0.05%) or H_2_O_2_ 300 μM for 2 h or a combined pretreatment with OLE 25 μM and H_2_O_2_ 300 μM for 24 and 2 h. **p < 0.01 and ***p < 0.0001 compared to vehicle.

Based on the results of the experimental setup a 24‐h pre‐incubation of differentiated AB1079 cells with 25 μM OLE followed by a 2‐h treatment with 300 μM H_2_O_2_ was tested (Figure 2A). As shown in Figure 2B cell viability was maintained at over 90% compared to vehicle after a 24‐h pretreatment with 25 μM OLE in combination with a 2‐h exposure to 300 μM H_2_O_2_ that was chosen for the subsequent analyses.

### 3.3 H_2_O_2_ ‐Induced ROS Formation Is Suppressed by OLE

**FIGURE 3:**
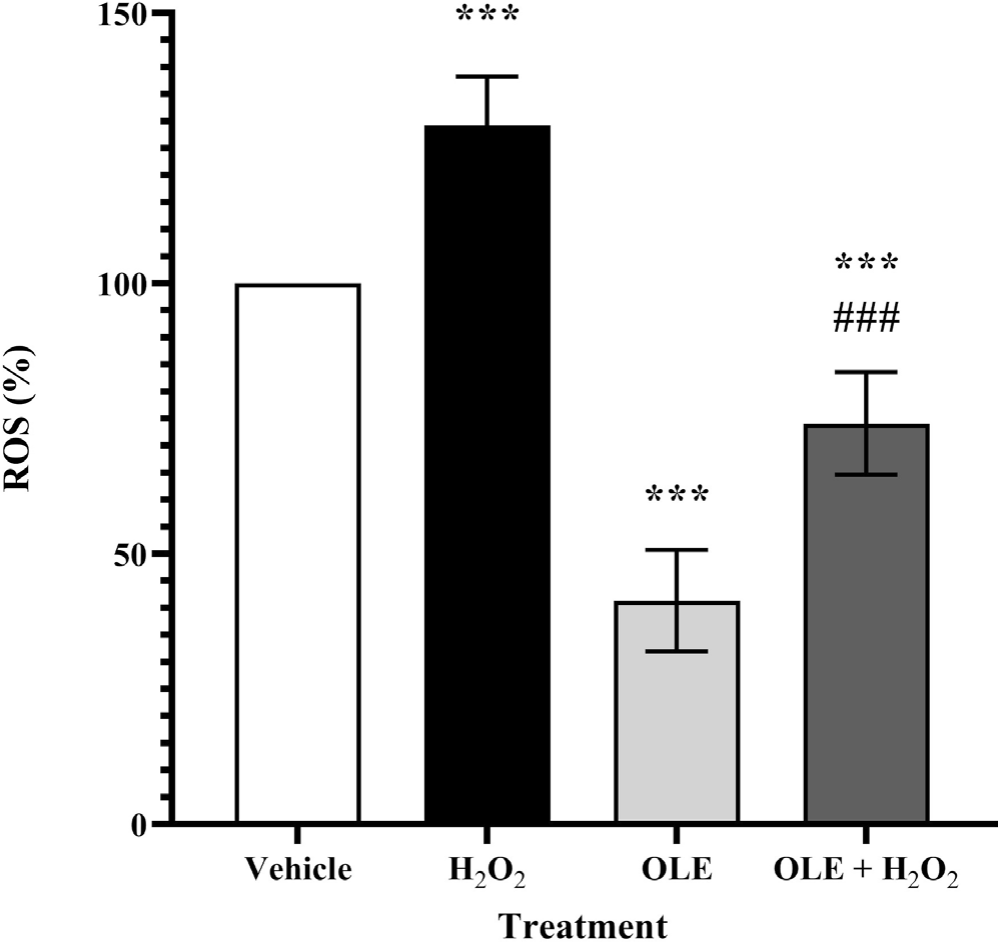
Analysis of ROS formation in AB1079 cells. Percentage of ROS in the vehicle and in the cells treated with H_2_O_2_, OLE or OLE + H_2_O_2_. Cells treated and pretreated with OLE show a significant decrease in ROS content, indicating an antioxidant effect. Vehicle, DMSO 0.05%; H_2_O_2_, 300 μM treated cells; OLE, 25 μM oleuropein‐treated cells; OLE + H_2_O_2_, 25 μM oleuropein‐pretreated and H_2_O_2_, 300 μM exposed cells. ***p < 0.0001 compared to vehicle; ^###^ *p* < 0.0001 compared to H_2_O_2_.

On the contrary, cells treated with OLE displayed a significantly reduced ROS amount (68%, *p* < 0.0001) compared to vehicle, suggesting a relevant antioxidant activity of OLE, which inhibits the endogenous production of ROS (Figure 3).

### 3.4 H_2_O_2_ ‐Induced Senescence Through ROS Formation Is Inhibited by OLE

**FIGURE 4:**
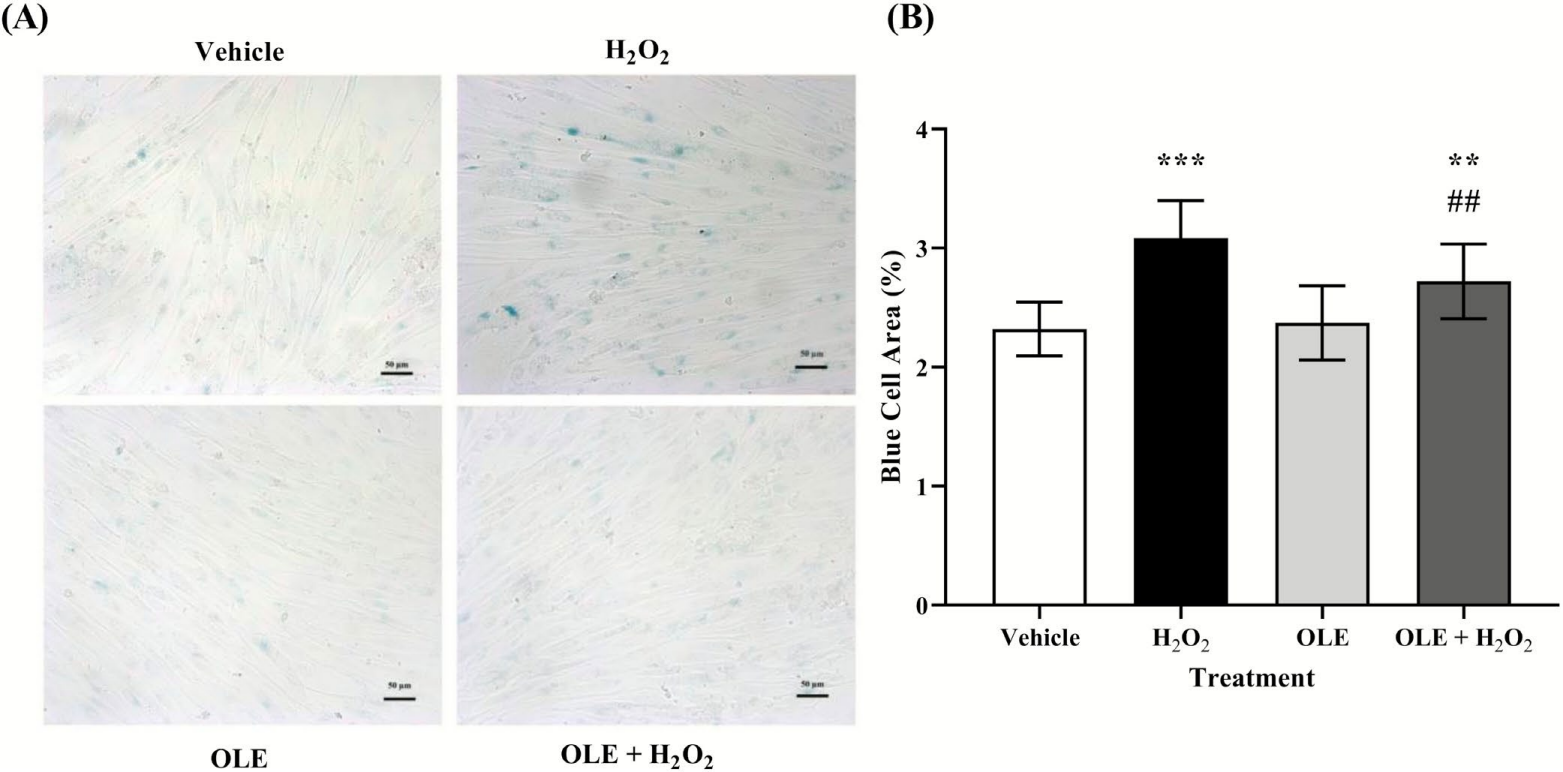
Analysis of cell senescence in AB1079 cells. (A) Representative images of SA‐β‐gal staining and the analysis of blue stained area in vehicle and in cells treated with H_2_O_2_, OLE or OLE + H_2_O_2_. Scale bar 50 μm. (B) OLE‐treated and pretreated cells show a significant decrease of stained blue area, suggesting a reduction of senescence cells. vehicle, DMSO 0.05%; H_2_O_2_ 300 μM treated cells; OLE, 25 μM oleuropein‐treated cells; OLE + H_2_O_2_, 25 μM oleuropein‐pretreated and H_2_O_2_ 300 μM exposed cells. **p < 0.01 compared to vehicle; ***p < 0.0001 compared to vehicle; ^##^ *p* < 0.001 compared to H_2_O_2_.

To determine whether exploiting the antioxidant properties of OLE could be a valid approach to attenuate the symptoms associated with senescence, the blue‐colored area indicating the presence of SA‐β‐gal was determined. As expected, Figure 4A shows that 24 h treatment with 25 μM OLE resulted in no change in SA‐β‐gal staining in AB1079 cells compared to vehicle. However, when the cells were pre‐treated with OLE and then exposed to H_2_O_2_ challenge, a significant reduction in blue stained area was observed (by 12%, *p* = 0.02) compared to H_2_O_2_‐treated cells (Figure 4B), confirming the protective role of OLE in preventing stress‐induced senescence in muscle cells.

**FIGURE 5:**
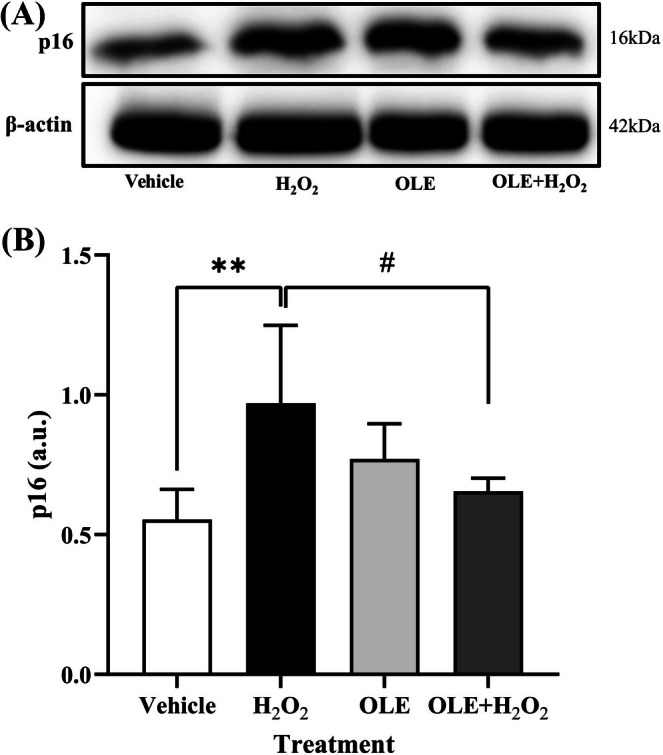
(A) Western blot analysis of p16 levels in vehicle, H_2_O_2_‐, OLE‐, and H_2_O_2_ + OLE‐treated cells. β‐actin was used as a loading control. (B) Densitometric data show the intensity of p16 normalized for β‐actin in control and treated cells. Vehicle, DMSO 0.05%; H_2_O_2_, 300 μM treated cells; OLE, 25 μM oleuropein‐treated cells; OLE + H_2_O_2_, 25 μM oleuropein‐pretreated and H_2_O_2_ 300 μM exposed cells. **p < 0.01 compared to vehicle; ^#^ *p* < 0.05 compared to H_2_O_2_.

To further confirm the senescence status of H_2_O_2_‐treated cells, the expression of the senescence marker p16 was examined by Western blot analysis (Figure 5A). As shown in Figure 5B, p16 protein levels were markedly increased in H_2_O_2_‐treated cells compared with the vehicle, indicating the induction of cellular senescence under oxidative stress. Notably, co‐treatment with OLE significantly reduced the level of p16 relative to H_2_O_2_‐treated cells, suggesting that OLE attenuates oxidative stress‐induced senescence in muscle cells. These findings are consistent with the SA‐β‐gal staining results and further support the anti‐senescent role of OLE. Although classical senescence markers such as SA‐β‐gal activity are typically assessed several days after stress induction, studies have shown that early oxidative stress–related responses, including p16 upregulation, can be detected within hours of H_2_O_2_ exposure particularly in models designed to evaluate the preventive effects of bioactive compounds [22].

### 3.5 OLE Increases the Gene Expression of the Antioxidant Defense System

**FIGURE 6:**
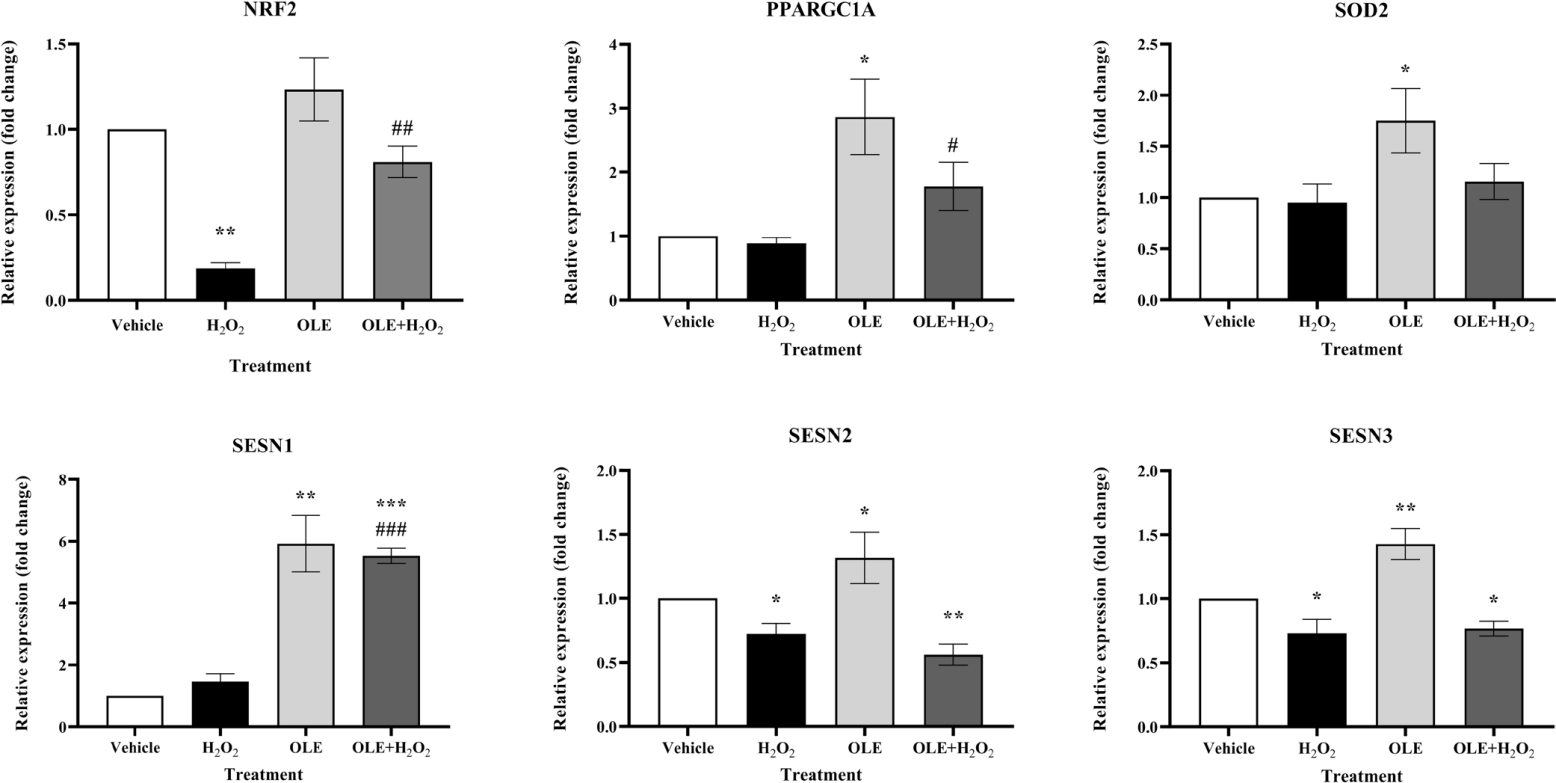
Expression of genes involved in antioxidant defense system. Gene expression analysis of NRF2, PPARGC1A, SOD2, SESN1, SESN2 and SESN3 in vehicle cells, H_2_O_2_‐, OLE‐ and OLE + H_2_O_2_‐treated cells. OLE treatment and pretreatment induce upregulation of the analyzed genes. This can explain the reduced ROS generation in cells treated and pretreated with OLE. Vehicle, DMSO 0.05%; H_2_O_2_, 300 μM treated cells; OLE, 25 μM oleuropein‐treated cells; OLE + H_2_O_2_, 25 μM oleuropein‐pretreated and H_2_O_2_ 300 μM exposed cells. *p < 0.05, **p < 0.01 and ***p < 0.0001 compared to vehicle; ^#^ *p* < 0.05, ^##^ *p* < 0.01 and ^###^ *p* < 0.0001 compared to H_2_O_2_.

It is well documented that natural antioxidant compounds have the ability to exert a direct free radical scavenging effect and/or indirect protection against oxidative stress stimuli by activating endogenous antioxidant defense systems in injured cells. To understand whether OLE can affect genes involved in endogenous defense mechanisms against oxidative stress in the proposed experimental design, the expression of NRF2, PPARGC1A, SOD2 and SESNs was investigated (Figure 6). In general, treatment with OLE led to a significant upregulation of all analyzed genes involved in the different steps of endogenous antioxidant defense. In particular, the expression of the genes PPARGC1A, SOD2, SESN1, SESN2 and SESN3 was increased by OLE treatment. In particular, SESN1 was strongly upregulated up to 5‐fold compared to the vehicle.

Conversely, acute exposure to H_2_O_2_ led to a decrease in the expression of NRF2, SESN2 and SESN3. H_2_O_2_ exposure almost completely abolished the expression of NRF2, a master regulator of downstream antioxidant genes, as well as SESN2.

It is interesting to note that the pretreatment of the cells with OLE and the subsequent H_2_O_2_ challenge led to a different pattern of results. Pretreatment with OLE clearly counteracted the detrimental effect of H_2_O_2_ on NRF2 gene expression, which in this case was dramatically upregulated compared to H_2_O_2_‐treated cells.

In a similar manner, the protective effect of OLE pretreatment resulted in a significant increase in the expression of PPARGC1A in response to H_2_O_2_ stimulus (Figure 6).

On the other hand, SOD2 gene expression, which hardly responded to oxidative stress anyway, could not maintain the enhancing effect observed with OLE treatment alone, but remained at a basal level after H_2_O_2_ exposure. In the case of sestrins, it should be noted that although a significant 2.77‐fold increase in the expression of the SESN1 gene was observed in cells pre‐treated with OLE and subsequently exposed to H_2_O_2_, confirming the specific behavior of OLE on this particular gene, the enhancing effect on the expression of the SESN2 and SESN3 genes induced by OLE treatment was impaired by the subsequent acute H_2_O_2_ treatment (Figure 6).

### 3.6 OLE Upregulates the Expression of Sestrin Proteins

**FIGURE 7:**
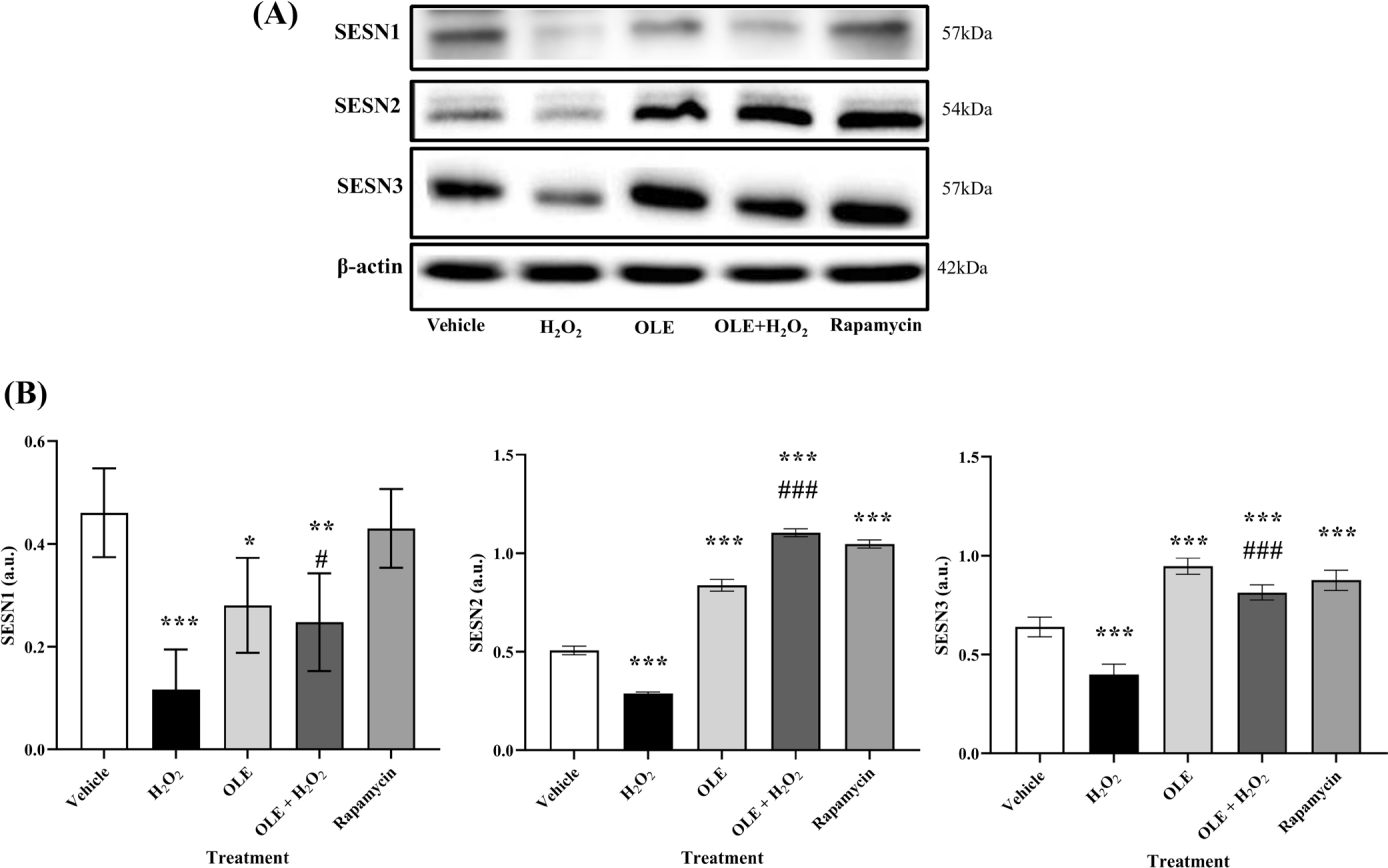
(A) The expression of sestrins was monitored by immunoblotting. β‐Actin was used as a loading control. (B) Densitometric data show the intensity of SESN1, SESN2 and SESN3 normalized for β‐actin in control and treated cells vehicle, DMSO 0.05%; H_2_O_2_, 300 μM treated cells; OLE, 25 μM oleuropein‐treated cells; OLE+H_2_O_2_, 25 μM oleuropein‐pretreated and 300 μM H_2_O_2_‐exposed cells; rapamycin, 500 nM rapamycin‐treated cells. *p < 0.05, **p < 0.01, ***p < 0.0001 compared to vehicle; ^#^ *p* < 0.05, ^###^ *p* < 0.0001 compared to H_2_O_2_.

Given the intriguing results on sestrins gene expression, a further analysis of protein translation in AB1079‐treated cells was performed (Figure 7A). It is important to emphasize that the protein expression of sestrins was consistent with the results obtained by RT‐PCR for their transcripts, with the exception of SESN1. Compared to vehicle, treatment with OLE led to a significant increase in protein expression, while H_2_O_2_ exposure led to a decrease in protein expression of SESN2 and SESN3. Unexpectedly, OLE pretreatment, which was ineffective in maintaining the efficacy of increasing SESN2 and SESN3 gene expression after H_2_O_2_ exposure, showed similar or even higher protein expression levels than in the absence of the oxidative stimulus. In contrast, both OLE and H_2_O_2_ treatment caused a significant down‐regulation of SESN1 protein expression compared to untreated cells, contrary to what RT‐PCR results previously suggested (Figure 7B). Nevertheless, pretreatment with OLE was able to compensate for the downregulation of SESN1 protein induced by H_2_O_2_ exposure, but, in any case, at a lower level than in the untreated cells. Interestingly, the results obtained in the differentiated AB1079 cells by treatment or pretreatment with OLE for SESN2 and SESN3 exactly matched those obtained with rapamycin, a known inducer of autophagy, which was used here as a positive control. This observation thus suggests a possible role of OLE in the upregulation of autophagy.

### 3.7 LC3B‐II Expression Confirms OLE‐Induced Autophagy

**FIGURE 8:**
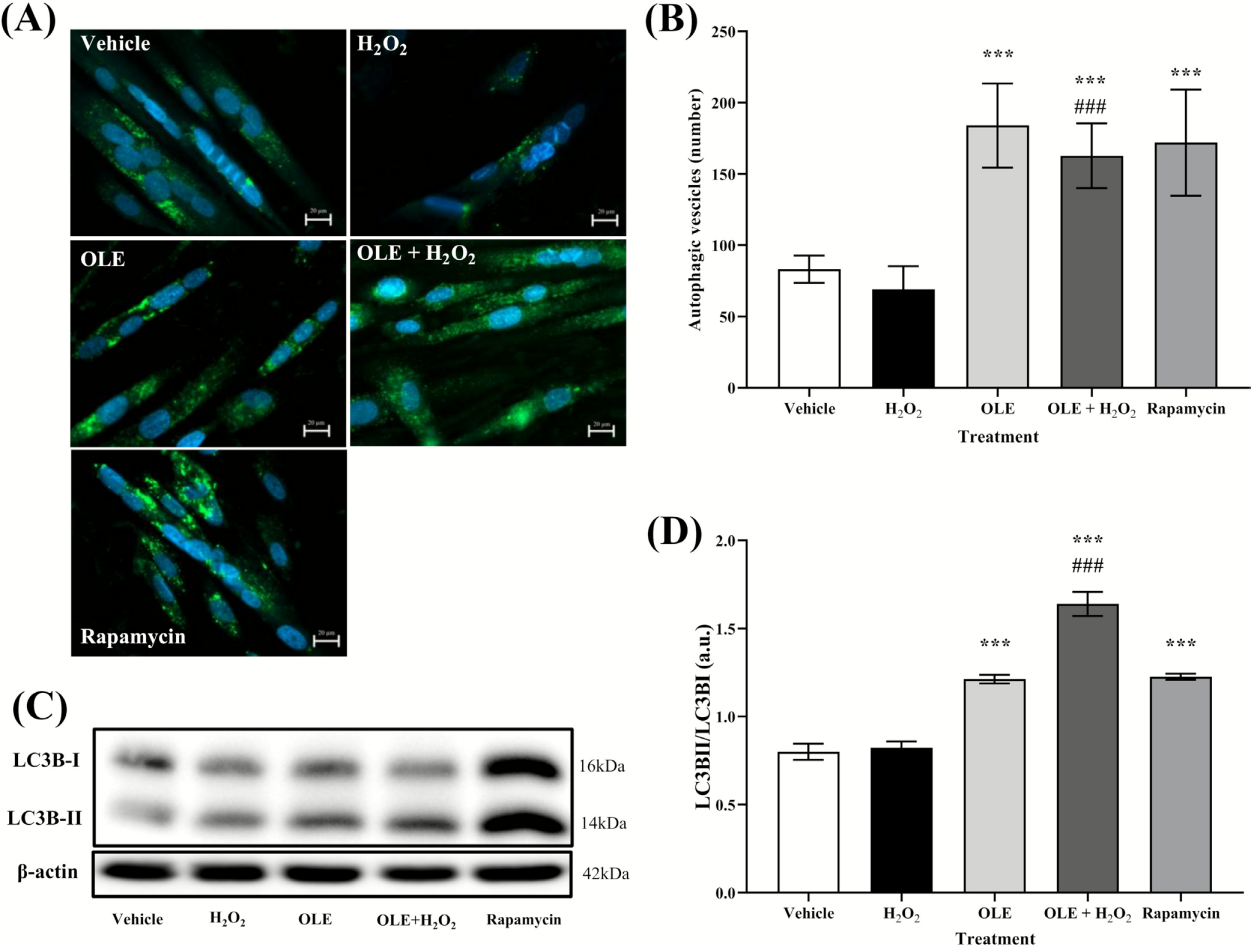
OLE increases autophagy and modulates the ratio of LC3B‐II/LC3B‐I in differentiated AB1079. (A) Representative images of Cyto‐ID fluorescent autophagy vesicles (green) and nuclear staining with Hoechst dye (blue) in AB1079 cells and in cells treated with H_2_O_2_, OLE, OLE + H_2_O_2_ and Rapamycin. Scale bar 20 μm. (B) Quantification of autophagic vesicles in vehicle and H_2_O_2_‐exposed cells, in OLE‐treated and pretreated cells and in cells treated with rapamycin used as positive autophagy control. As in the Rapamycin, OLE treatment and pre‐stimulation significantly increased the number of autophagic vesicles compared to vehicle and H_2_O_2_ exposed cells. (C) Western blot analysis of LC3B‐I and LC3B‐II. (D) Quantification of the ratio between LC3B‐II and LC3B‐I. β‐actin was used as a loading control. Vehicle, DMSO 0.05%; H_2_O_2_, 300 μM treated cells; OLE, 25 μM oleuropein‐treated cells; OLE+H_2_O_2_, 25 μM oleuropein‐pretreated and 300 μM H_2_O_2_‐exposed cells; rapamycin, 500 nM rapamycin‐treated cells. ***p < 0.0001 in comparison to vehicle; ^###^ *p* < 0.0001 in comparison to H_2_O_2_.

There is increasing evidence of a link between impaired or reduced autophagy and the aging processes [33]. To investigate the relationship between the anti‐aging effect of OLE and the induction of autophagy, the structure of autophagic vesicles in differentiated AB1079 cells was detected using the fluorescent dye Cyto‐ID (Figure 8A). This dye specifically labels autophagic compartments, while lysosomes and endosomes are only minimally stained [34]. Remarkably, in the differentiated AB1079 cells, the number of autophagic vesicles was found to be significantly increased in both the OLE‐treated and pre‐treated cells and in the rapamycin‐treated positive control compared to the vehicle and the H_2_O_2_‐treated cells, respectively. This observation confirmed the stimulatory effect of OLE on the autophagy process (Figure 8B).

To validate the ability of OLE to induce autophagy, the protein expression levels of a canonical autophagy marker, namely microtubule‐associated protein 1A/1B light chain 3 (LC3), were also determined (Figure 8C). The ratio of LC3B‐II to LC3B‐I expression was significantly higher in cells incubated with OLE alone and in cells pre‐incubated with OLE and subsequently treated with H_2_O_2_ compared to vehicle and H_2_O_2_‐exposed cells, respectively (Figure 8D). It is important to note that an increase in the ratio of LC3B‐II to LC3B‐I generally indicates increased autophagy activity. Evidence for this phenomenon was the observation that cells treated with the autophagy inducer rapamycin exhibited an LC3B‐II/LC3B‐I ratio comparable to that observed upon treatment with OLE.

**FIGURE 9:**
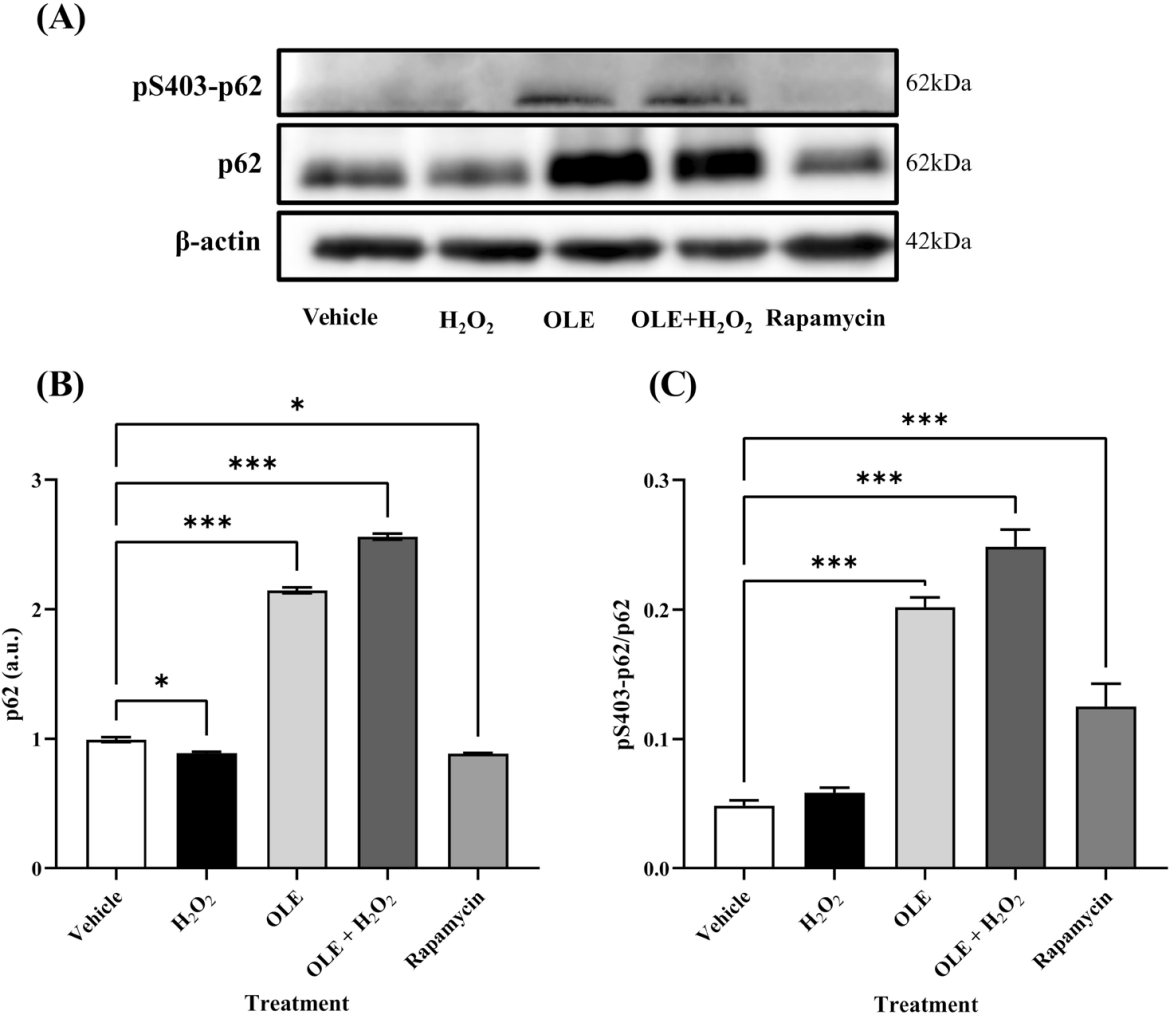
(A) Western blot analysis of pSer403‐p62, p62 expression in vehicle‐, H_2_O_2_‐, OLE‐, and H_2_O_2_ + OLE‐treated muscle cells. β‐actin was used as a loading control. (B) Quantitative analysis of p62 band intensity. (C) Quantitative analysis of the pSer403‐p62/p62 ratio band intensity. Vehicle, DMSO 0.05%; H_2_O_2_, 300 μM treated cells; OLE, 25 μM oleuropein‐treated cells; OLE + H_2_O_2_, 25 μM oleuropein‐pretreated and 300 μM H_2_O_2_‐exposed cells; rapamycin, 500 nM rapamycin‐treated cells. *p < 0.05, ***p < 0.001 in comparison to vehicle. OLE + H_2_O_2_‐treated cells also differed from H_2_O_2_‐treated cells for *p* < 0.05 (significance does not show).

To further investigate the role of autophagy in the cellular response to OLE and oxidative stress, we analyzed the expression of the autophagy adaptor protein p62 and its phosphorylated form at Ser403 by Western blot (Figure 9A). The results showed p62 levels differed significantly in all treated cells compared with the vehicle control. These results must be interpreted together with the LC3B ratio data and indicate that H_2_O_2_ treatment did not activate autophagy, although there was a significant decrease in p62 levels compared to the vehicle. In contrast, OLE treatment, either alone or combined with H_2_O_2_, increased p62 levels and the LC3B ratio relative to the vehicle and to H_2_O_2_, suggesting that OLE modulated autophagy dynamics. Differently, rapamycin promoted an increase in the LC3B ratio and a decrease in the p62 level and therefore led to complete autophagy (Figure 9B). Treated cells with OLE, OLE + H_2_O_2_ and rapamycin showed significantly different levels of the pSer403‐p62 from the vehicle (Figure 9C). These findings support the hypothesis that OLE regulates autophagy to maintain cellular homeostasis under stress conditions.

### 3.8 The Activation of AMPKα During OLE‐Induced Autophagy is Time‐Dependent

**FIGURE 10:**
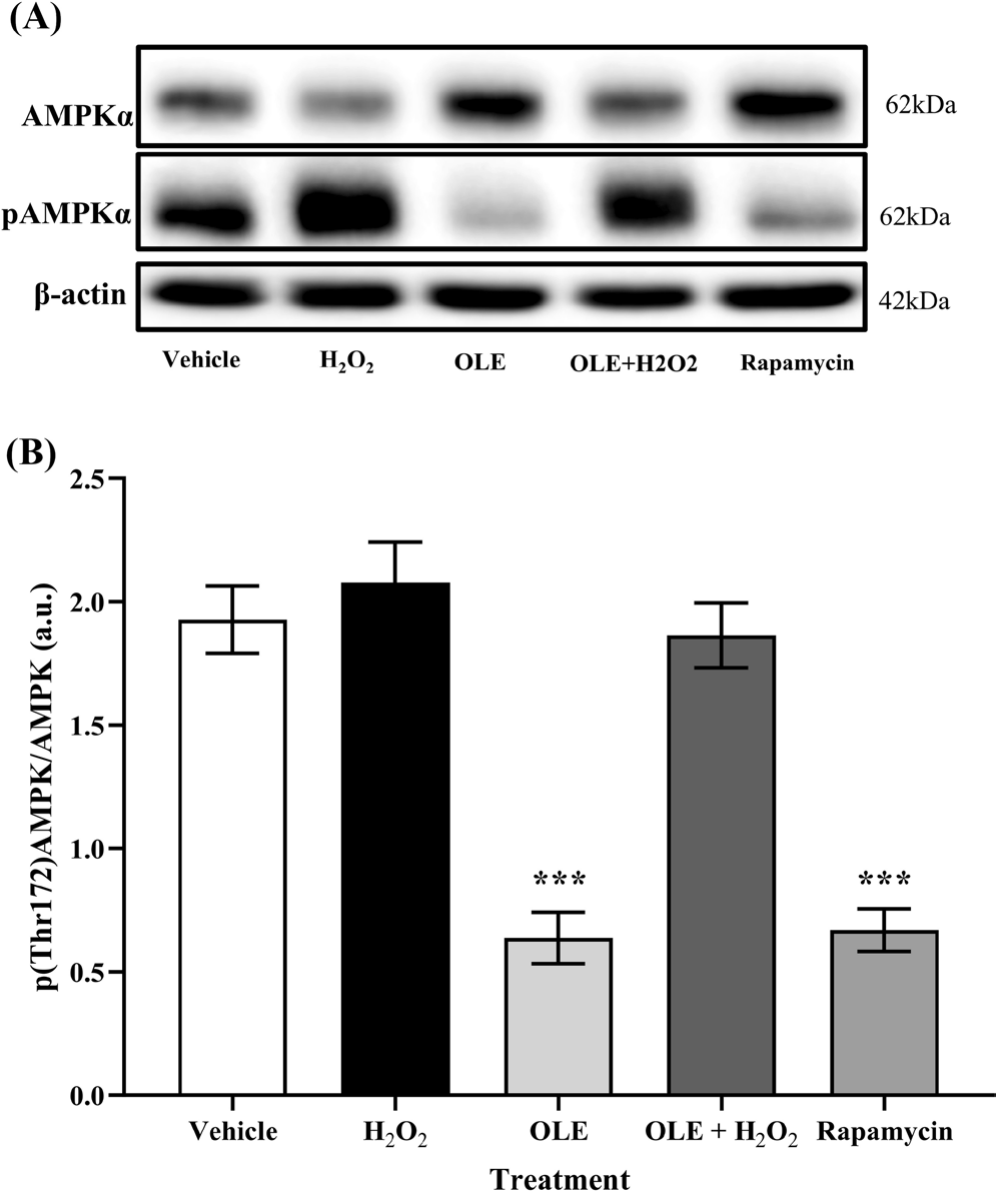
Quantitative expression of AMPKα phosphorylated at threonine 172. (A) Western blot analysis of total AMPKα and AMPKα phosphorylated at threonine 172. (B) Quantification of the ratio between AMPKα phosphorylated at threonine 172 and total AMPKα. β‐Actin was used as a loading control. Vehicle, DMSO 0.05%; H_2_O_2_ 300 μM treated cells; OLE, 25 μM oleuropein‐treated cells; OLE + H_2_O_2_, 25 μM oleuropein‐pretreated and 300 μM H_2_O_2_‐exposed cells; rapamycin, 500 nM rapamycin‐treated cells. ***p < 0.0001 compared to vehicle.

To better understand the relationship between OLE treatment and autophagy activation in the AB1079 experimental model, the phosphorylation of threonine 172 (pThr172‐AMPKα), which is the regulatory trigger for AMPKα activation, was monitored (Figure 10A). The results revealed a surprising finding: in differentiated AB1079 cells treated with OLE alone and in cells treated with rapamycin, the level of T172 phosphorylation was significantly lower than in vehicle and H_2_O_2_‐exposed cells (Figure 10B).

**FIGURE 11:**
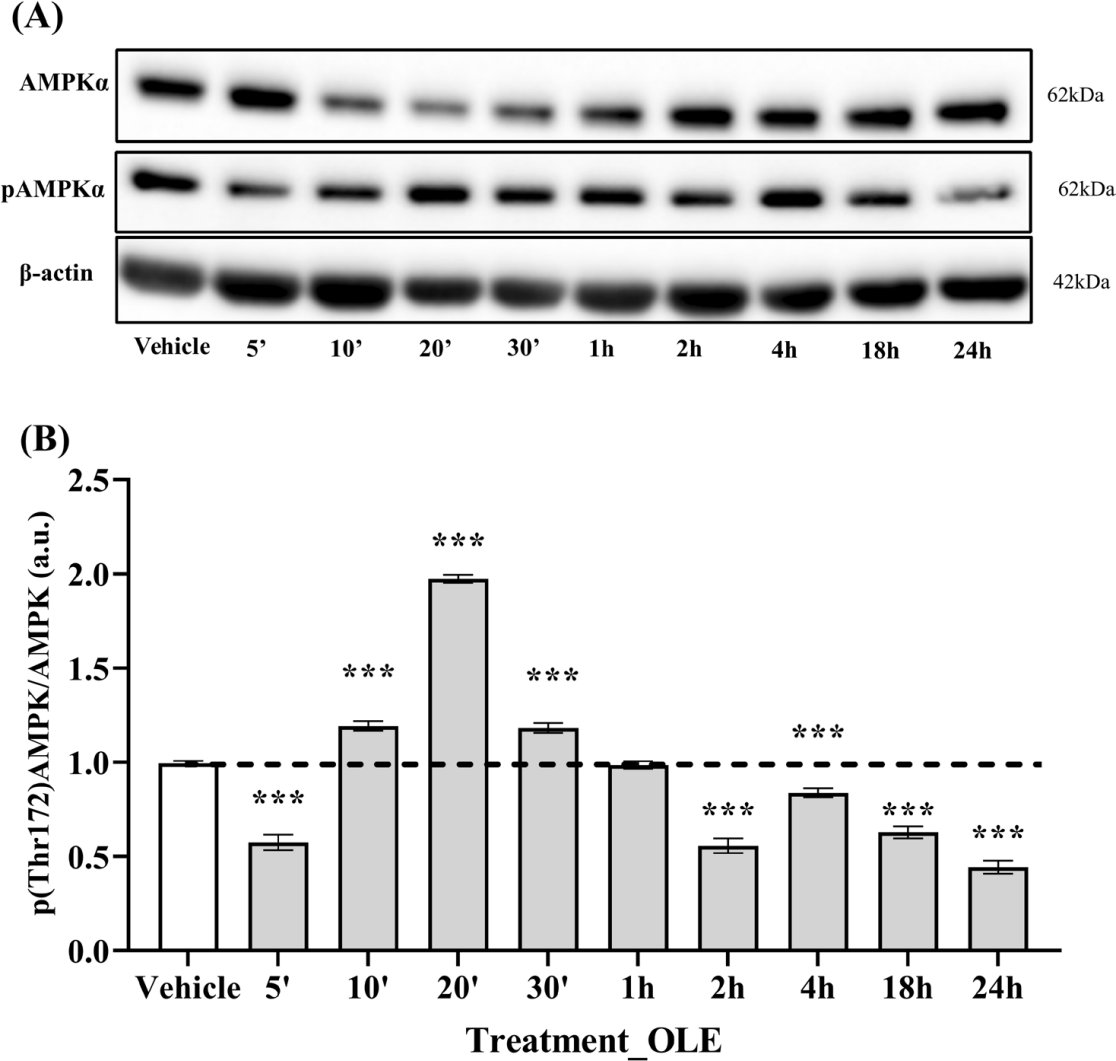
Time course of treatment with OLE showed an oscillatory expression of active AMPKα in AB1079 cells. (A) Immunoblot of AMPKα phosphorylated at threonine 172 and total AMPKα during short time points of treatment with OLE. (B) Quantitative ratio between pAMPKα and total AMPKα protein at different time points of OLE treatment. OLE, 25 μM oleuropein‐treated cells; ***p < 0.0001 compared to vehicle.

Low AMPKα phosphorylation rates at this site are generally associated with its inactive form. However, given the increased ratio of LC3B‐II to LC3B‐I previously observed in cells treated with OLE for 24 h as well as in the rapamycin‐treated positive control, we investigated whether the hypothesized OLE‐mediated AMPK activation might instead represent an early event that declines after 24 h of cell treatment. Accordingly, treatment with OLE was performed at different time points and the presence of activated AMPK was detected by immunoblotting (Figure 11A). Remarkably, phosphorylated AMPK was highest after 20 min of cell treatment (i.e., 97% more than in untreated cells) and then decreased below the phosphorylation level of the control sample to a minimum after 24 h of incubation (Figure 11B).

**FIGURE 12:**
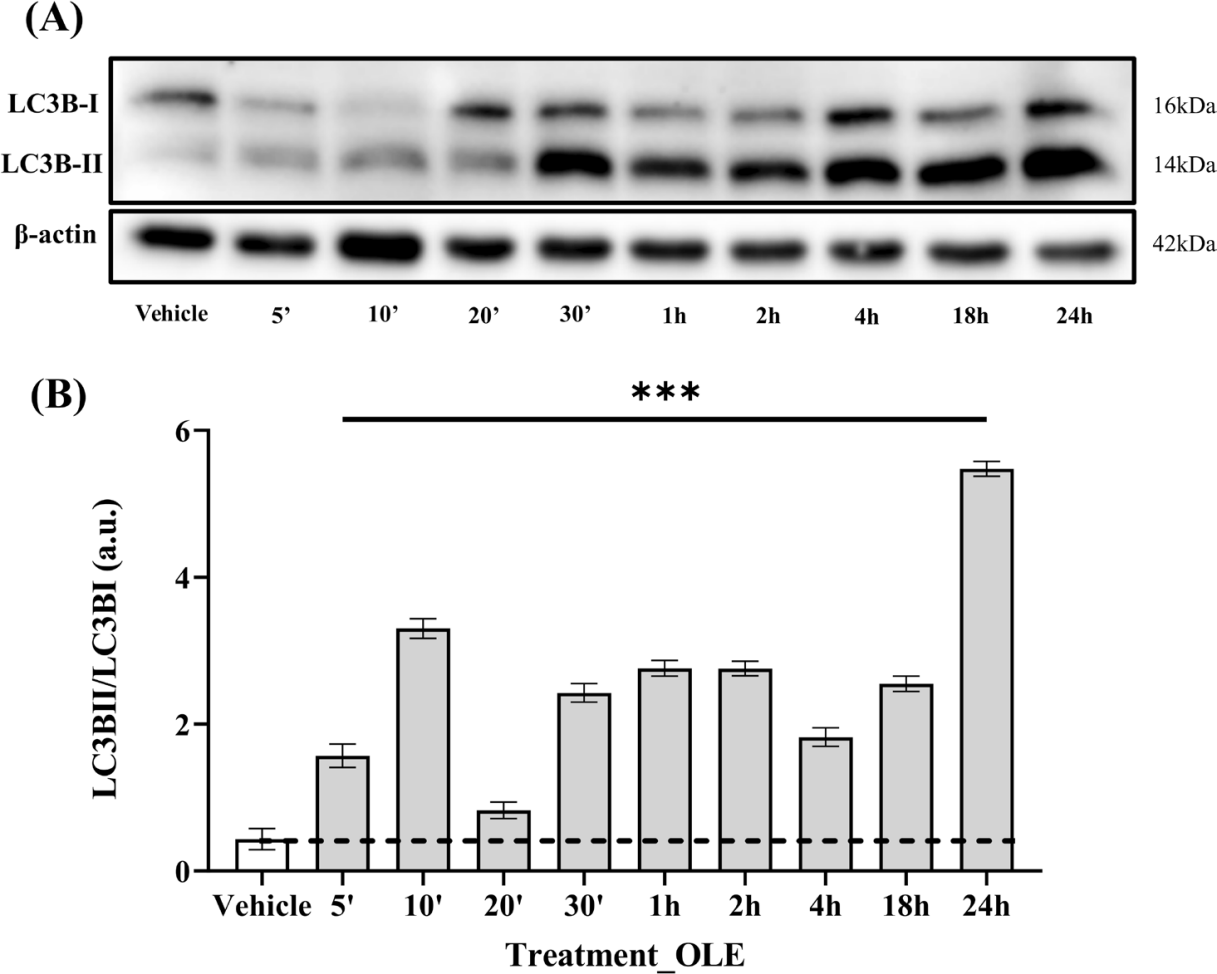
Expression of active LC3B‐II/LC3B‐I ratio in AB1079 cells during treatment with OLE over 24 h. (A) Immunoblot of LC3B‐II and LC3B‐I during short time points of treatment with OLE. (B) Quantitative ratio between LC3B‐II/LC3B‐I protein at different time points of OLE treatment. OLE, 25 μM oleuropein‐treated cells; ***p < 0.0001 compared to vehicle.

In addition, the expression of LC3B was monitored simultaneously and interestingly, the most significant increase in the ratio of LC3B‐II to LC3B‐I was observed after 24 h of OLE treatment, showing an oscillating trend, as with pThr172‐AMPKα, but with an opposite tendency (Figure 12).

### 3.9 FOXO3a was Influenced by OLE via AMPK After 24 h

**FIGURE 13:**
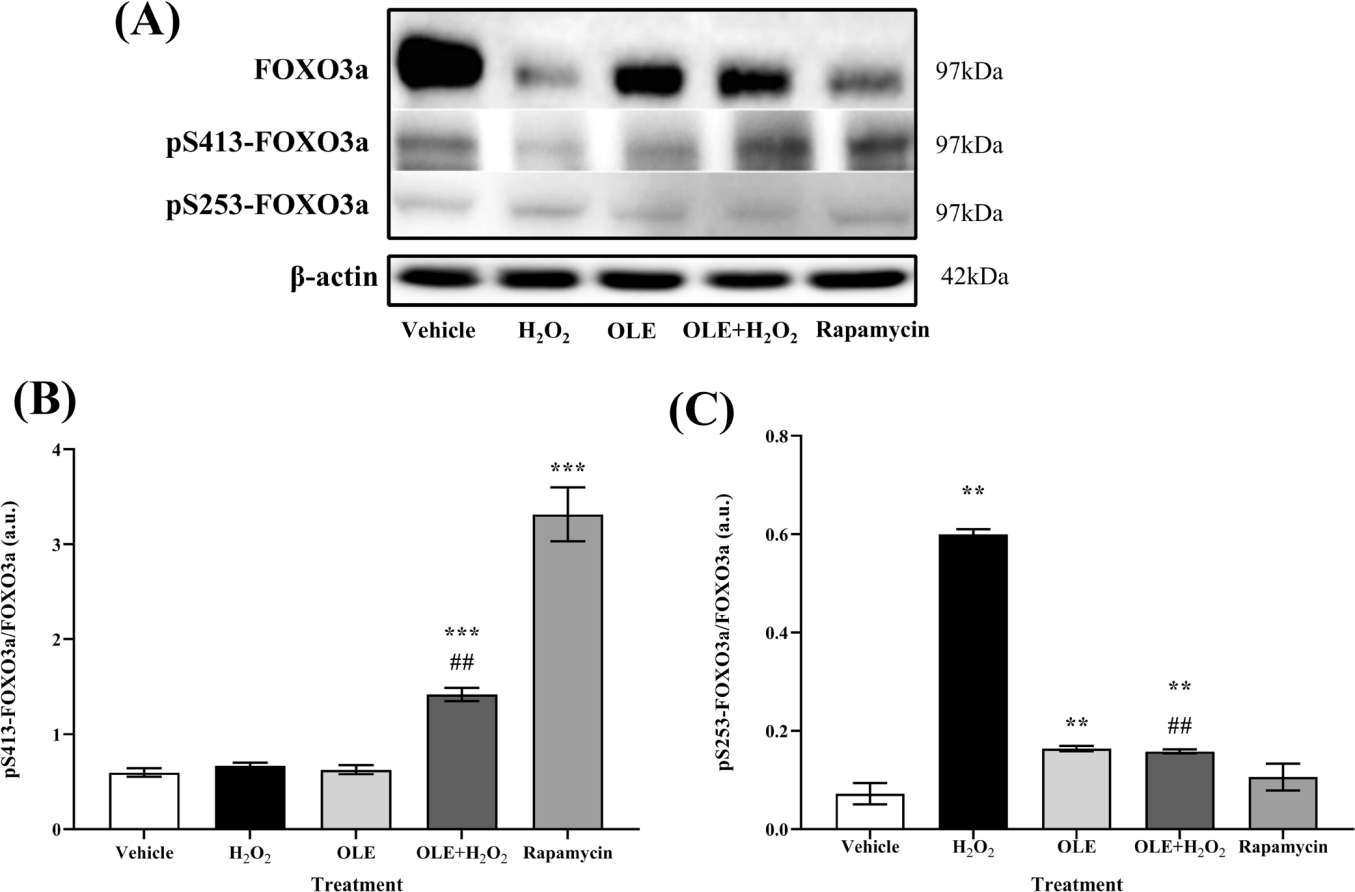
Effects of H_2_O_2_ and OLE on phosphorylation and expression of FOXO3a. (A) Western blot analysis of total FOXO3a, FOXO3a phosphorylated at residue Ser 413 and at residue Ser 253. (B) Immunoblotting analyses of the ratio between total FOXO3a and FOXO3a phosphorylated by AMPK at residue Ser 413 (C) Immunoblotting analyses of the ratio between total FOXO3a and FOXO3a phosphorylated by AKT at residue Ser 253. Vehicle, DMSO 0.05%; H_2_O_2_, 300 μM treated cells; OLE, 25 μM oleuropein‐treated cells; OLE+H_2_O_2_, 25 μM oleuropein‐pretreated and 300 μM H_2_O_2_‐exposed cells; rapamycin, 500 nM rapamycin‐treated cells. **p < 0.01, ***p < 0.0001 compared to vehicle; ^##^ *p* < 0.01 compared to H_2_O_2_.

Finally, the potential effect of OLE treatment on FOXO3a, an important regulator of cell homeostasis, stress response and longevity, was investigated (Figure 13). Together with other post‐translational modifications, phosphorylation at different specific sites is responsible for controlling the subcellular localisation and transcriptional activity of FOXO3a. Indeed, AMPK can phosphorylate FOXO3a at Ser413, which increases its transcriptional activity without affecting its subcellular localisation [16]. Conversely, phosphorylation at Ser253 by PKB/AKT has been shown to lead to the exclusion of FOXO3a from the nucleus and subsequent association with 14–3‐3 proteins, which in turn inhibits its transactivation activity [35]. The phosphorylation levels of Ser253 and Ser413 were evaluated as a ratio to the expression of total FOXO3a (Figure 13A). In the AB1079 cell model, treatment with OLE had no effect on the phosphorylation levels of FOXO3a at Ser413 compared to vehicle, but pretreatment with OLE led to an increase in the phosphorylation levels of both FOXO3a residues compared to untreated cells and only at pSer413 compared to H_2_O_2_ (Figure 13B). In addition, the phosphorylation level of Ser253 decreased compared to H_2_O_2_. As expected, treatment with rapamycin led to a significant increase (*p* < 0.0001) in the phosphorylation levels of Ser413 but not of Ser253 (Figure 13C). H_2_O_2_ stimulation led to a strong increase in pSer253, while no significant difference was observed in pSer413 compared to vehicle (Figure 13).

The phosphorylation levels at both residues were influenced by the total protein content of FOXO3a, which differed between treatments and especially after H_2_O_2_ and rapamycin treatments. The assessment of the two phosphoresidues revealed that pretreatment with OLE had a significant effect on acute H_2_O_2_ exposure.

## 4 Discussion

The benefits of the Mediterranean diet are often attributed to the high content of polyphenols in olives and olive oil, which have been shown to protect against various diseases, including age‐related diseases [36]. Muscle diseases such as sarcopenia, muscle weakness and atrophy are associated with metabolic changes in skeletal muscle cells, often due to oxidative damage, which in turn disrupts cell homeostasis. In fact, increased production of ROS has been shown to have a significant impact on cell function and overall muscle health. ROS are by‐products of normal cellular metabolism, particularly during aerobic respiration [37]. While low to moderate levels of ROS play a crucial role in cell signaling and homeostasis, excessive ROS can lead to oxidative stress and damage to cellular components such as DNA, proteins and lipids [38]. Oxidative stress affects muscle function in two ways: it can lead to muscle fatigue and promote muscle wasting [39]. In this context, it has been shown that this impairment is of particular importance during periods of intense physical exertion, in advanced age and in connection with various myopathies [40]. The human body is equipped with complex and redundant antioxidant defense mechanisms, including a number of enzymes such as superoxide dismutase, catalase and glutathione peroxidase, which help to neutralize excess ROS and protect cells from oxidative damage. Maintaining a balance between ROS production and antioxidant defense is essential for optimal muscle health and function. Therefore, the management of oxidative stress through an appropriate diet, regular exercise and possibly the intake of antioxidants could be a strategic approach to counteract the most debilitating effects of aging [41].

In the present study, oleuropein aglycone, the major phenolic compound in extra virgin olive oil, was shown to enhance the expression of genes involved in the antioxidant defense system and to promote the expression of key transcription factors that regulate H_2_O_2_‐induced oxidative stress, including NRF2, PPARGC1A and FOXO3a, in the myoblast cell line AB1079. Furthermore, our results suggest that oleuropein exerts a positive regulatory influence on the autophagic process as well as on the expression of the stress‐induced metabolic regulators SESN2 and SESN3.

Numerous research studies have shown that H_2_O_2_ triggers oxidative stress in cells, which leads to cell damage, apoptosis and the promotion of cell senescence [11, 42]. Elevated H_2_O_2_ concentrations have been shown to induce premature senescence, and it has been established that H_2_O_2_ treatment can be considered a standard procedure to simulate this condition [27, 31]. For this reason, H_2_O_2_ was used in differentiated AB1079 myoblasts to create a muscle cell model of oxidative stress by increasing intracellular ROS levels and promoting stress‐induced senescence without greatly affecting cell viability (Figure 1). This optimized in vitro model was used to analyze the preventive effect of OLE. It should be emphasized that this protocol detects early markers of stress‐induced senescence following acute H_2_O_2_ exposure and does not recapitulate the full establishment of chronic cellular senescence, which typically requires longer post‐treatment culture periods. This limitation should be considered when interpreting present results. Moreover, it is important to note that the effects of OLE may vary depending on cell type, duration and concentration of exposure [43]. In general, OLE is considered preventive rather than therapeutic [8], as recently shown in the differentiated canine Myok9 cell line [11]. In accordance with the experimental design, incubation of AB1079 cells with 25 μM OLE for 24 h prior to 2‐h acute exposure to H_2_O_2_ (300 μM) showed 91% cell viability (Figure 2), accompanied by a significant decrease (43%) in ROS formation (Figure 3) and an approximate 12% reduction in cellular SA‐β‐gal blue area and in p16 level.

It is noteworthy that oleuropein exerts its antioxidant effect via various mechanisms that enhance free radical scavenging, as reported by Visioli et al. [9]. However, oleuropein can also modulate cellular signaling processes by enhancing signal transduction pathways against oxidative stress. In this regard, both 24‐h OLE treatment and pretreatment induced the expression of NRF2, PPARGC1A, SOD2 and SESN1 genes in the human muscle cell model AB1079. The transcription factor NRF2 has been shown to activate the transcription of antioxidant enzyme genes [44], and overexpression of PPARGC1A has been shown to promote the PGC‐1ɑ pathway of mitochondrial biogenesis, thereby reducing oxidative damage by regulating cellular oxidant‐antioxidant homeostasis in skeletal muscle [45]. In addition, upregulation of NRF2 and PPARGC1A has been associated with the induction of SOD2 expression, as previously described in rats [46] and SESN2 [47]. It should be highlighted that although H_2_O_2_ induced the formation of ROS, it did not significantly alter the expression of SOD. Consequently, the dose used and the timing of the experimental setup were sufficient to induce oxidative stress without stimulating catalase‐mediated detoxification mechanisms [48]. This finding was confirmed by the reduction of NRF2 expression in human cells treated with H_2_O_2_ compared to vehicle.

SESNs play an important role in combating aging by reducing oxidative stress and improving cell metabolism. Indeed, SESNs have been shown to delay the progression of age‐related metabolic diseases by regulating their redox activity and the AMPK/mTORC1 metabolic pathway [49]. In this context, the combined antioxidant effect of several polyphenols and SESNs has already been demonstrated [50, 51, 52], suggesting that both SESNs and OLE‐induced signaling pathways converge in the AMPK signaling cascade. OLE exerted a remarkable effect on the transcription of SESN1, confirming its response to oxidative stress stimuli. However, at the protein level, only SESN2 and SESN3 were significantly upregulated, suggesting that different post‐transcriptional regulatory modifications or protein stability mechanisms are involved. It should be noted that the 25 μM OLE concentration used here exceeds physiologically achievable plasma levels but was selected as the highest non‐cytotoxic dose in vitro and is consistent with previous mechanistic studies. Thus, these results provide cellular insights, while in vivo relevance remains to be validated at physiological concentrations and with OLE metabolites. Bae et al. [53] hypothesized that the antioxidant effect of SESN2 is promoted by the degradation of Keap1 via p62‐dependent autophagy, which in turn activates Nrf2. On the other hand, FOXO3a‐induced SESN3 expression activates AMPK, which in turn inhibits mTORC1, promoting autophagy and thereby maintaining cellular health [54]. It is worth noting that the antioxidant properties of OLE and its ability to activate key signaling pathways via AMPK may contribute to the modulation of FOXO3a activity. This in turn may help to protect cells from oxidative damage and promote healthy aging. In addition, the role of AMPK in oxidative stress resistance is linked to the activating phosphorylation of the transcription factor FOXO3a. Upon phosphorylation, FOXO promotes the transcription of antioxidant genes, including those encoding superoxide dismutase, catalase and sestrins [55].

In our experimental model, the preventive effect of OLE was related to the promotion of activation of autophagy, as shown by the significant increase in the number of autophagic vesicles in both OLE‐treated and pretreated cells compared to vehicle cells and H_2_O_2_‐treated cells. The involvement of autophagosome formation was confirmed by the increased ratio of LC3B‐II to LC3B‐I and by the ratio of the phosphorylated form at Ser403 of p62 to total p62 in OLE‐ and OLE + H_2_O_2_‐treated cells. It must be considered that during autophagy, the isolation membrane is recruited to the sequestosome by LC3‐p62 interaction, leading to autophagosome growth [56]. Moreover, Ro et al. [57] demonstrated that the stress‐inducible sestrin 2 promotes ULK1‐dependent phosphorylation of p62. The significantly higher levels of sestrin 2 and the phosphorylated form of p62 in OLE‐ and OLE + H_2_O_2_‐treated cells are in agreement with these findings and indicate that OLE promotes an autophagic response, supporting cell survival under oxidative conditions, without completing the process, as observed in rapamycin‐treated cells. In fact, only rapamycin treatment showed a decrease in total p62 level, indicating that the autophagic flux was completed.

It is known that the induction of autophagy is generally associated with AMPK activation and that OLE induces autophagy via the AMPK/mTOR signaling pathway [57]. However, in differentiated AB1079 cells treated with OLE, the phosphorylation level of AMPK at Thr172 was significantly lower than in vehicle and H_2_O_2_‐exposed cells. We therefore investigated whether OLE promotes the autophagic process by inducing AMPK activity for shorter incubation periods. To confirm this hypothesis, phosphorylated AMPK was significantly increased after only 20 min of OLE exposure (i.e., about 97% more than in vehicle cells) and then significantly decreased and was 55% lower than in vehicle after 24 h. Remarkably, during the preparation of this manuscript, another study reported that autophagy induction by AMPK can exhibit oscillatory behavior [58].

In fact, autophagy is not a continuously active process but switches on and off in response to the current state of the cell. The oscillatory nature of autophagy is controlled by a series of interconnected feedback loops within the mTORC1‐AMPK‐ULK1 regulatory network. This periodic activation and inactivation of autophagy would ensure that the cells can adapt to different stress conditions [58, 59, 60]. In addition, Kazyken et al. [61] showed that LC3B is not affected in the absence of AMPK, but that its lipidation increases under these conditions, as we demonstrated in our experimental model by analyzing the ratio of LC3B‐II/LC3B‐I over 24 h of treatment with OLE.

It should be noted that rapamycin can also induce biphasic phosphorylation of AMPK in cancer cells [62] and in mouse C2C12 myotubes, where this biphasic response is due to the sequential disruption of mTORC1 and mTORC2 complexes [63, 64].

Finally, the interaction between AMPK, AKT and FOXO3a occurs via a complex network of cellular signaling in which AMPK and AKT play opposing roles, with important implications for the regulation of cell survival, stress resistance, apoptosis and autophagy in response to exercise [54, 65]. Indeed, the FOXO‐inducible gene SESN3 inhibits ROS accumulation, while AKT stimulates ROS production through FOXO‐dependent downregulation of Sesn3 [54]. Despite these observations in mouse embryonic fibroblasts (MEF cells), this is consistent with the observed increase in pSer253‐FOXO3a and downregulation of SESN3 in cells treated with H_2_O_2_. Cells pre‐treated with OLE and subsequently exposed to H_2_O_2_ showed their potential by activating AMPK, which led to a strong increase in pSer413‐FOXO3a and a significant decrease in pSer253‐FOXO3a compared to H_2_O_2_.

## 5 Conclusion

The results of this study support the hypothesis that OLE reduces oxidative stress and activates antioxidant and autophagy‐related signaling in human myoblasts in vitro. These findings suggest a potential role in cellular processes linked to aging. However, future studies using physiological concentrations, metabolites, and in vivo models are necessary before translating these observations into clinical applications.

## Authors’ Contributions

**Giulia Polacchini:** performed the experiments and analyses; analyzed data and interpreted results; edited and revised the manuscript. **Andrea Venerando:** performed the experiments; analyzed data and interpreted results; edited and revised the manuscript. **Anne Bigot:** edited and revised the manuscript. **Monica Colitti:** conceived and designed research, analyzed data and interpreted results, drafted the manuscript; edited and revised the manuscript. All authors read and approved the final manuscript.

## Ethics Statement

The authors have nothing to report.

## Conflicts of Interest

The authors declare no conflicts of interest.

## Data Availability

The data that support the findings of this study are available from the corresponding author upon reasonable request.
